# The ongoing need for local services for people with complex mental health problems

**DOI:** 10.1192/pb.bp.114.048470

**Published:** 2014-12

**Authors:** Helen Killaspy

**Affiliations:** 1 University College London, London

## Abstract

Despite developments in mental healthcare over recent decades, there remains a group of people with very complex needs who require lengthy admissions and high levels of support in the community on discharge. This is the group that mental health rehabilitation services focus on. In the context of contemporary mental health services that minimise in-patient lengths of stay, the needs of this group must not be overlooked. Providing a local, ‘whole system, integrated rehabilitation care pathway’ requires intelligent commissioning in order to avoid the social exclusion of this group to the ‘virtual asylum’ of out-of-area placements.

## Introduction

Despite the many developments in mental health treatments and investment in community-based services over the past few decades, some people continue to present with particularly complex problems that require longer-term care. In the UK, mental health rehabilitation services focus on this group. Simply put, these are the people who have not recovered adequately to be discharged home. The majority have a diagnosis of psychosis complicated by treatment refractory symptoms, severe negative symptoms and cognitive impairments (particularly dysexecutive problems). Many have coexisting mental health problems (including common mental disorders, substance misuse problems and/or premorbid conditions such as mild learning disability or Asperger syndrome) and most have physical health problems as a result of a combination of poor diet, lack of exercise, smoking and the side-effects of psychotropic medication. As a consequence of these multiple issues, everyday functioning tends to be severely impaired and challenging behaviours and poor engagement are common.^1^

This group represent a relatively small proportion of people in receipt of mental healthcare; it is estimated that around 14% of people newly diagnosed with psychosis will develop the kinds of complex problems that will require rehabilitation services.^2^ However, they usually require lengthy in-patient admissions and more intensively supported accommodation on discharge, which results in them absorbing 25–50% of the mental health and social care budget.^3^ They can therefore be considered a ‘low volume, high needs’ group.

Most people are referred to in-patient rehabilitation services when the admitting service has exhausted treatment options (around 80% of referrals come from acute admission wards). However, the mean length of admission prior to transfer to rehabilitation services is around 10 months,^4^ which may suggest that acute services are keen to exhaust therapeutic possibilities before referral or, perhaps more likely, that there is often a long wait for a rehabilitation bed because of underprovision. The remaining 20% of referrals come from forensic/secure services,^4^ illustrating the importance of local rehabilitation services in the pathway out of secure services.

## The rehabilitation care pathway

Rehabilitation services operate as a ‘whole system, integrated care pathway’ across in-patient and community settings provided by statutory and non-statutory health and social care sectors.^5^ This pathway includes different types of in-patient rehabilitation units and a range of supported accommodation services for people to be discharged to. Almost all NHS mental health trusts in England provide in-patient rehabilitation services.^4^ These include: ‘high dependency units’ that are usually sited within a hospital setting, with an expected length of stay of up to 2 years and where most patients are involuntarily detained; ‘community rehabilitation units’, the majority of which can also take detained patients and have an expected length of stay of up to 3 years; and ‘complex care units’ for those who require a longer period of in-patient care that are usually hospital based, take detained patients and have an expected length of stay of over 5 years. The private sector also provides in-patient rehabilitation units, usually with a similar specification to high dependency units or with a higher level of security and staffing. These were previously referred to as ‘low secure rehabilitation units’ but now tend to be called ‘locked rehabilitation’ to differentiate them from forensic services (as a result of new commissioning arrangements for the latter). Some offer specialist facilities for people with especially challenging behaviours and less common combinations of complex problems. Supported accommodation services are usually provided by the independent or voluntary sector and include 24 h staffed residential/nursing care homes, individual or shared staffed tenancies and floating outreach services providing support to those living in independent tenancies. Over half the NHS mental health trusts in England have a community rehabilitation team^4^ that provides clinical input to local supported accommodation services to enable patients in their ongoing recovery and progress through the care pathway.^6^ For those without a community rehabilitation team, clinical input is provided by other community mental health services such as community mental health teams, recovery teams or assertive outreach teams.

## Effectiveness of current provision

Although the mental health rehabilitation care pathway has been well described,^7^ there has been relatively little research into its effectiveness. We previously surveyed 140 patients of mental health rehabilitation services in one inner London trust and found that those in complex care units had much higher needs and poorer social functioning than those in high dependency and community-based rehabilitation units, and those in high dependency and complex care units had more severe challenging behaviours than those in community units.^8^ This cohort was followed up after 5 years, by which time two-thirds had achieved and sustained successful community discharge and 9% had achieved fully independent living. The only factor found to be associated with positive progress along the pathway was medication adherence.^9^ In Ireland, a prospective case–control study compared outcomes over 18 months for people with complex needs receiving mental health rehabilitation services and those awaiting them. Those receiving rehabilitation services were eight times more likely to achieve and sustain community discharge and there was a greater improvement in social functioning. Those with higher levels of unmet need, substance misuse and challenging behaviours were less likely to achieve successful community living.^10^

In this issue of *The Psychiatric Bulletin*, Meaden and colleagues describe a survey of over 100 patients of ten NHS mental health rehabilitation units in the West Midlands (two high dependency units, five community rehabilitation units and three complex care units).^11^ Their results illustrate the high level of challenging behaviours among these patients and the difficulties of engaging them in treatment. Their findings concur with those of previous studies; there were higher levels of aggressive challenging behaviours among those in the high dependency units and more socially unacceptable challenging behaviours among those in the complex care units, whereas those in the community rehabilitation units were better engaged with their rehabilitation and presented with fewer challenging behaviours. The data are cross-sectional and causality cannot be inferred, but they suggest that these different components of the pathway are necessary to support people at different stages of their recovery.

The results also highlight the importance of, and difficulty in, maintaining therapeutic optimism when working with people with such complex needs. Meaden et al acknowledge the need to build on the rapport between patients and their primary nurse to enable the person to engage in rehabilitative interventions that involve other members of the team and they suggest approaches to facilitate this. Staff working in these settings need to hold hope for patients who have often been considered ‘untreatable’ in other parts of the mental health system and who may have lost a sense of their own identity, purpose and connections with family and friends through the course of their illness.^12^ Holding therapeutic optimism is no easy task. Staff need to receive training to develop an understanding of the process of individual recovery and how to support this.^13,14^ They need to hold in mind the long-term view for the gradual improvement of symptoms and everyday functioning that does come about for the majority of those who receive rehabilitation, that enables them to leave hospital and move to the community successfully without further relapse, readmission or placement breakdown. Working with people who improve at a relatively slow rate requires patience and commitment. Most staff work in only one component of the rehabilitation pathway and it can be difficult to hold on to that longer-term view. In addition, the metric of success in contemporary in-patient settings is an average length of stay that is as short as possible. Understanding the need for longer-term treatment of the complex needs group is vital and making the case for local rehabilitation services is therefore a key skill for rehabilitation practitioners.^15^

## Importance of local rehabilitation services

We already know what happens if we do not invest in local mental health rehabilitation services; those with complex needs become stuck on acute in-patient units and are eventually relocated to out-of-area placements in private hospitals, nursing and residential care homes where they often remain out of sight and out of mind in a ‘virtual asylum’.^16^ This phenomena has been widely reported in deinstitutionalised countries^17^ and heavily criticised.^18–20^ In 2011, the Royal College of Psychiatrists published a helpful guide for commissioners in England and Wales on best practice in the delivery of services for people with complex needs to address this problem that called for ongoing investment in services that make up the local rehabilitation care pathway.^21^ However, with recent changes in commissioning and commissioners in most areas of England, this guidance has probably been at best forgotten and at worst ignored. With the imminent introduction of a tariff-based system of mental healthcare, it is imperative that commissioners and senior managers understand the need for appropriate investment in local specialist mental health rehabilitation services and supported accommodation for those with complex needs if we are to avoid a repeat of the shameful exportation of this group into out-of-area placements that lack a rehabilitative ethos and compound the social dislocation of this vulnerable group.^18–20^

Two years ago, the UK Schizophrenia Commission called for investment in high-quality services to deliver evidence-based treatments for people with long-term psychosis.^22^ The evidence base for mental health rehabilitation services is growing. We now know that the majority of people receiving rehabilitation are able to achieve and sustain successful community living and that improving the quality of care in in-patient rehabilitation units promotes patient autonomy, the main aim of rehabilitation.^4^ Studies like that of Meaden and colleagues^11^ are important in furthering our understanding of the needs of this group. In addition, two national programmes of research are underway across England, both funded by the National Institute of Health Research, to investigate the aspects of care delivered in different components of the rehabilitation pathway that are most beneficial (the Rehabilitation Effectiveness and Activities for Life (REAL) study and the Quality and Effectiveness of Supported Tenancies (QuEST) study). We hope that these programmes will help to guide future practice and ensure appropriate investment in the treatment, care and support for those with the most complex mental health needs.
